# Epidemiological and pathological study of feline morbillivirus infection in domestic cats in Japan

**DOI:** 10.1186/s12917-016-0853-y

**Published:** 2016-10-11

**Authors:** Eun-Sil Park, Michio Suzuki, Masanobu Kimura, Hiroshi Mizutani, Ryuichi Saito, Nami Kubota, Youko Hasuike, Jungo Okajima, Hidemi Kasai, Yuko Sato, Noriko Nakajima, Keiji Maruyama, Koichi Imaoka, Shigeru Morikawa

**Affiliations:** 1 Department of Veterinary Science, National Institute of Infectious Diseases, Tokyo, 162-8640 Japan; 2 Tokyo Metropolitan Animal Care and Consultation Center, Jounanjima Branch Office, Tokyo, 143-0002 Japan; 3 Department of Pathology, National Institute of Infectious Diseases, Tokyo, 162-8640 Japan

**Keywords:** Feline morbillivirus, Cat, Kidney disease, Epidemiology, Inflammation

## Abstract

**Background:**

Feline morbillivirus (FmoPV) is a novel paramyxovirus found to infect domestic cats. FmoPV has been isolated in several countries in Asia and Europe and is considered to have genetic diversity. Also, it is suspected to be associated with feline renal diseases including tubulointerstitial nephritis (TIN), which affects domestic cats with a high incidence rate.

**Results:**

To clarify the state of FmoPV infection among domestic cats in Japan, an epidemiological survey was conducted. Twenty-one out of 100 cats were found to have serum antibodies (Ab) against FmoPV-N protein by indirect immunofluorescence assay (IF) using FmoPV-N protein-expressing HeLa cells. Twenty-two of the cats were positive for FmoPV RNA in the urine and/or renal tissues. In total, 29 cats were positive for Ab and/or viral RNA. These FmoPV-infected cats were classified into three different phases of infection: RNA+/Ab + (14 cats), RNA+/Ab- (8 cats) and RNA-/Ab + (7 cats). In immunohistochemistry (IHC), 19 out of 29 cats were positive for FmoPV-N protein in kidney tissues; however, the FmoPV-N protein was located in the inflammatory lesions with severe grade in only four out of the 19 cats. Since 15 out of 29 infected cats were positive for viral RNA and Ab, approximately half of the infected cats were persistently infected with FmoPV.

**Conclusions:**

A statistically significant difference was observed between infection of FmoPV and the presence of inflammatory changes in renal lesions, indicating a relationship between FmoPV infection and feline renal diseases. However, we could not obtain histopathological evidence of a relationship between FmoPV infection and TIN.

## Background

A novel paramyxovirus, feline morbillivirus (FmoPV), has recently been detected in domestic cats [1–8]. FmoPV is genetically most closely related to viruses such as canine distemper virus (CDV), measles virus (MV), rinderpest virus (RPV), peste-des-petits-ruminants virus (PPRV), phocine distemper virus (PDV) and cetacean morbillivirus (CMV), belonging to the genus morbillivirus in the family *Paramyxoviridae* [1–5]. FmoPV showed genetic diversity among isolates [3–5], and a natural recombination in the envelope protein region between viruses in different clades was also found [4]. In Germany, three groups of feline paramyxoviruses (FPaV) have been detected, and these were associated with feline chronic kidney diseases (CKD) including lower urinary tract diseases (LUTD) [5]. Phylogenetically, the first group of these viruses belongs to the same cluster of FmoPV with 99 % homology, whereas the second group represents a new cluster between FmoPV and other morbilliviruses. The third group represents a group that is distinct from FmoPV and other morbilliviruses. A seroepidemiological survey of CDV infection in Asian countries showed that domestic cats were susceptible to CDV infection, but CDV was not virulent in domestic cats [9]. At the moment, it is not yet confirmed that FmoPV is classified in the genus morbillivirus or in a novel genus separate from the genus morbillivirus.

Kidney failure is one of the most important and common diseases in domestic cats. It can be divided into acute kidney disease (AKD) and chronic kidney disease (CKD), or inherent kidney disease and acquired kidney disease [10–13]. AKD, which could be caused by toxins, trauma, infection, shock, blockage of the blood flow and heart failure [11], is reversible and can affect cats of all ages. CKD affects domestic cats, especially middle-aged or older cats [14], and its prevalence increases according to age, affecting up to half of cats older than 15 years [14]. CKD could result from infection, blockages, dental disease, high blood pressure and cancer. In particular, idiopathic CKD such as pyelonephritis, glomerulonephritis and chronic tubulointerstitial nephritis (TIN) due to unknown causes has been reported extensively [10, 11, 15–17]. It is suspected that FmoPV is one of the causative agents of CKD [1, 5], such as chronic TIN. Therefore, it is important to clarify the characteristics or the pathogenicity of FmoPV and the pathogenesis in domestic cats as the natural host. In this respect, large-scale epidemiological investigation is considered to be indispensable.

In this study, epidemiological and pathological studies were performed to demonstrate the seroprevalence of FmoPV and the relationship between FmoPV and CKD in Japan. These studies revealed that the infection rate of FmoPV was considerable and FmoPV might be related to urinary tract diseases.

## Results

### Detection of FmoPV by RT-PCR and phylogenetic analysis

Cat urine and renal tissues were examined for the presence of FmoPV RNA by nested RT-PCR [2]. Seventeen cats (17 %) were positive for FmoPV RNA in urine, and 18 cats (18 %) were positive in renal tissues (Table 1). Among these cats, 13 cats (13 %) were both positive in urine and tissues. Four cats (4 %) were positive in the urine but negative in the tissues, whereas five cats (5 %) were negative in the urine but positive in the tissues.

**Table 1:** Detection of FmoPV RNA by RT-PCR

		In urine (*n* = 100)	
		-	+
In kidney tissues (*n* = 100)	-	78	4
	+	5	13

**Fig. 1:**
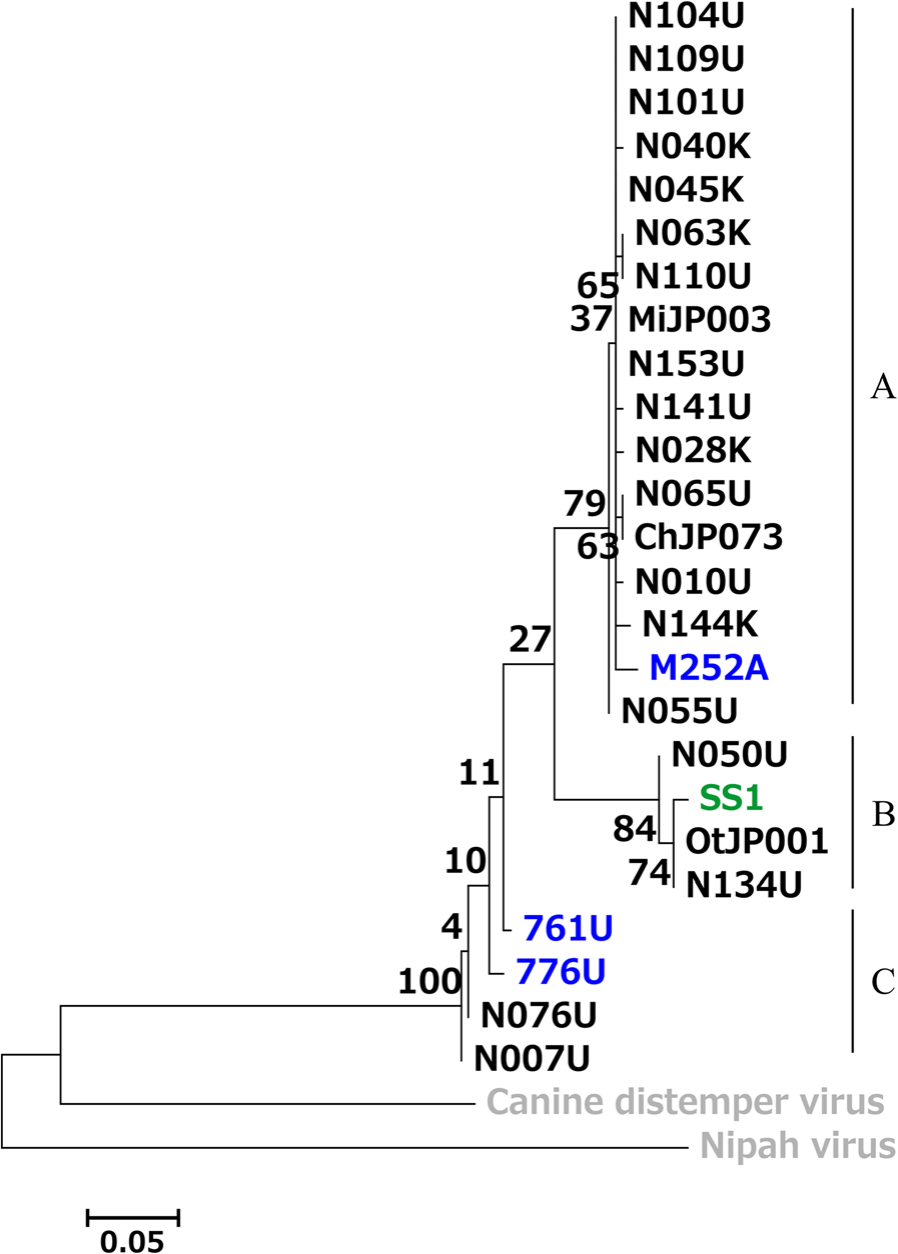
Phylogenetic analysis based on partial L protein sequences of FmoPV in infected cats in Tokyo. Phylogenic tree was inferred using the maximum likelihood method in the MEGA6 package. The percentage of replicate tree in which the associated taxa clustered together in the bootstrap test (1000 replicates) is shown next to the branches. The tree is drawn to scale, and the scale bar indicates the branch length corresponding to 0.05 substitutions per site. The strains from Hong Kong and Japan (Kyoto) are represented by *blue* and *green*, respectively. Nucleotide sequences of CDV and Nipah virus are used as outgroups and represented in *gray*. Information about the strain names and accession numbers is listed in Table 2. Statistically significant differences (*P* < 0.01) were obtained in the distribution of the pairwise distances of clusters A, B and C

Nucleotide sequences of all the PCR-positive samples were analyzed phylogenetically with those of FmoPVs in Hong Kong and Japan (Kyoto), and CDV and Nipah virus as outgroups. They were divided into three groups (Fig. 1). To determine the groups of FmoPV strains in the phylogenetic tree are stable, pairwise distances were compared by histogram by the method described previously [18]. Statistically significant differences (*P* < 0.01) were obtained in the distribution of the pairwise distances of clusters A, B and C (data not shown). FmoPV strains from Hong Kong and some strains of Japan were grouped into the same clusters, cluster A and C, thus a phylogenetic relationship with geographic distribution of FmoPV in two countries was not observed (Fig. 1).

### Detection of antibodies against FmoPV-N protein by Immunofluorescence (IF) test

**Fig. 2:**
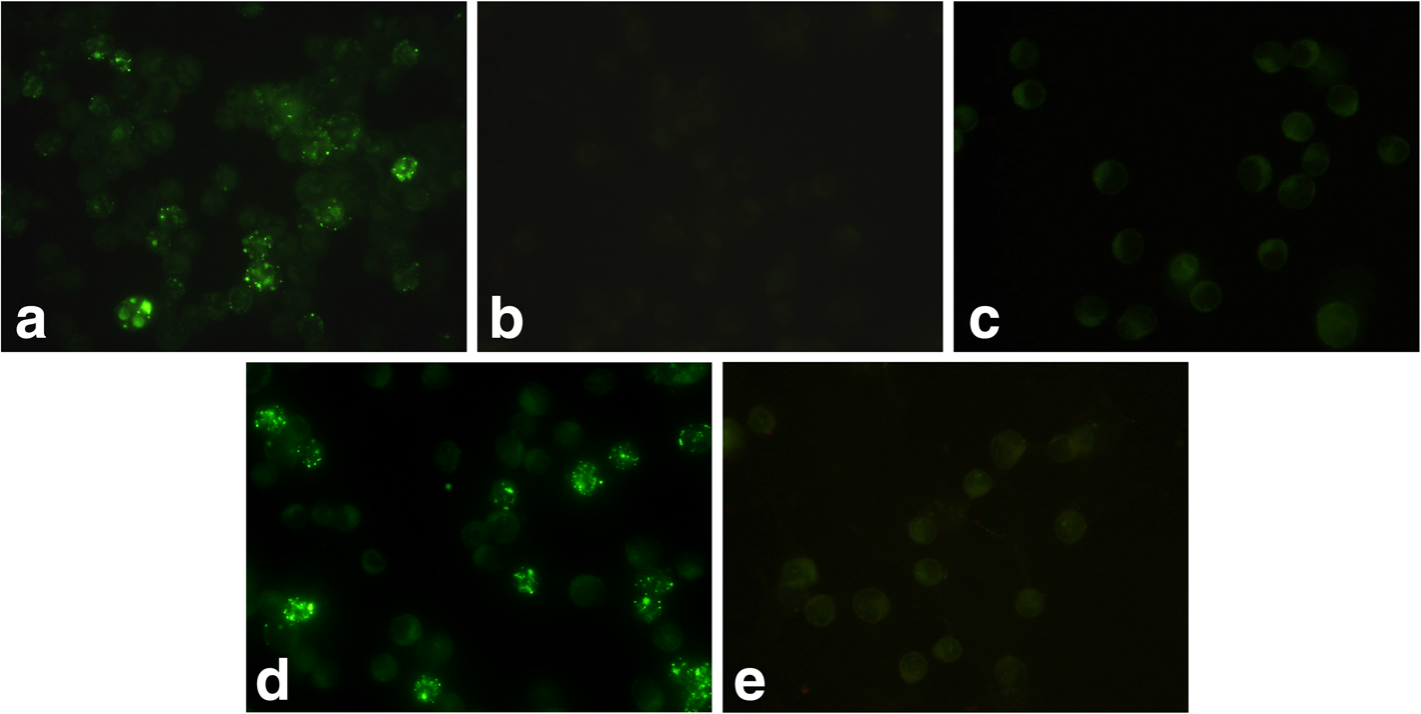
IF staining. **a** FmoPV-N immunized rabbit serum strongly reacted to FmoPV-N expressing HeLa cells, but did not react to empty vector, pKS336, transfected HeLa cells (**b**), nor to mock HeLa cells (**c**). FmoPV antibody positive cat (N045, 1:40) reacted to FmoPV-N expressing HeLa cells (**d**), but did not react to mock HeLa cells (**e**). Granular *apple green* signal was observed in the cytoplasm, represented by FITC

To detect FmoPV-N antibodies by IF test, FmoPV-N expressing HeLa cells were prepared. The specificity of the IF test was confirmed by serum from rabbit immunized with the purified FmoPV-N and its pre-immune negative control rabbit serum. As shown in Fig. 2, FmoPV-N immunized rabbit serum reacted to FmoPV-N expressing HeLa, but not to mock-HeLa cells nor empty plasmid, pKS336, transfected HeLa cells. Negative control rabbit serum did not react to any of the HeLa cells. Specificity of the IF test was also confirmed using FmoPV antibody positive cat serum (N045) (Fig. 2). Thus, all the cat sera were tested by the IF test to detect antibodies against FmoPV-N.

**Fig. 3:**
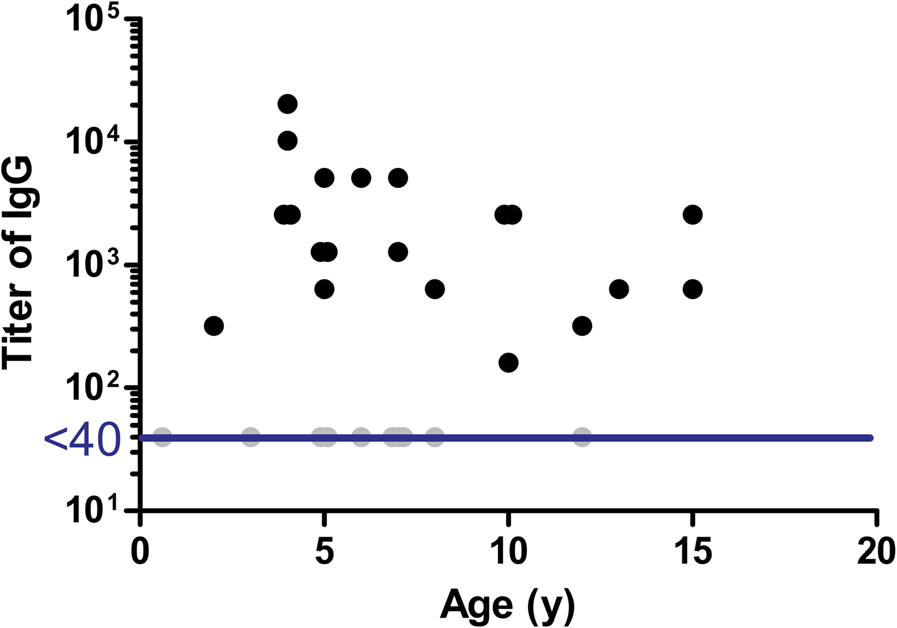
The relationship between age of FmoPV-infected cats and Ab titer. Ab titers in IF test are represented by *black dots*. Sera showing Ab titers of less than 1:40 are considered negative (*gray dots*)

Twenty-one percent of cats had Ab against FmoPV-N protein (Table 2 and Fig. 2). Ab titers ranged between 1:160 to 1:20,480 or above. The Ab titers appeared to be high in middle-aged cats (from 4 to 8 years old, Fig. 3), although not significantly. All the FmoPV antibody positive cat sera were tested negative by IF test using CDV-N expressing HeLa cells (data not shown), indicating there is no serological cross reactivity between CDV-N and FmoPV-N at least by IF test. There were four patterns: RNA+/Ab + (14 cats), RNA+/Ab- (eight cats) and RNA-/Ab + (seven cats) (Table 2) and RNA-/Ab- (71 cats, data not shown). In total, 29 out of 100 cats (29 %) were positive for Ab and/or viral RNA, thus they were considered to be FmoPV-infected. Fourteen out of 29 cats were positive for viral RNA and Ab, indicating that approximately half of the infected cats were persistently infected with FmoPV.

**Table 2:** Detection of viral RNA, IgG and antigen

No. of cases	ID of cat	Degree of lesions	FmoPV RNA		Antibody titer in IF	IHC	Accession no.	Strain
			urine	tissue				
1^a^	N010	++	P^1^	P	>20,480	***	LC080842	N010U
2^a^	N101	++	P	P	5120	***	LC080851	N101U
3^a^	N153	+++	P	P	640	***	LC080858	N153U
4	N007	+	P	P	10,240	**	LC080841	N007U
5	N110	+	P	P	1280	**	LC080854	N110U
6	N134	++	P	P	160	**	LC080855	N134U
7	N141	+	P	P	2560	*	LC080856	N141U
8	N020	+	P	N^2^	5120	*		
9	N050	+++	P	N	640	**	LC080846	N050U
10	N104	none	P	N	1280	**	LC080852	N104U
11	N028	+++	N	P	5120	**	LC080843	N028K
12	N040	++	N	P	1280	**	LC080844	N040K
13†	N055	++	P	P	<40	***	LC080847	N055U
14	N001	++	P	P	<40	**	AB924120	OtJP001
15	N003	+	P	P	<40	**	AB924121	MiJP003
16	N073	+++	P	P	<40	*	AB924122	ChJP073
17	N076	none	P	P	<40	**	LC080850	N076U
18	N109	++	P	P	<40	*	LC080853	N109U
19	N140	+	N	N	640	*		
20	N045	++	N	P	2560	none	LC080845	N045K
21	N144	+++	N	P	640	none	LC080857	N144K
22	N063	+	N	P	<40	none	LC080848	N063K
23	N065	+	P	N	<40	none	LC080849	N065U
24	N024	+	N	N	2560	none		
25	N064	+++	N	N	2560	none		
26	N131	+	N	N	320	none		
27	N136	++	N	N	640	none		
28	N138	none	N	N	320	none		
29	N148	++	N	N	2560	none		

^a^ IHC-positive in inflammatory lesions

1 positive

2 negative

+ mild

++ moderate

+++ severe

* mild

** moderate

*** extensive

### Histopathological examination of FmoPV infection in cat renal tissues

**Fig. 4:**
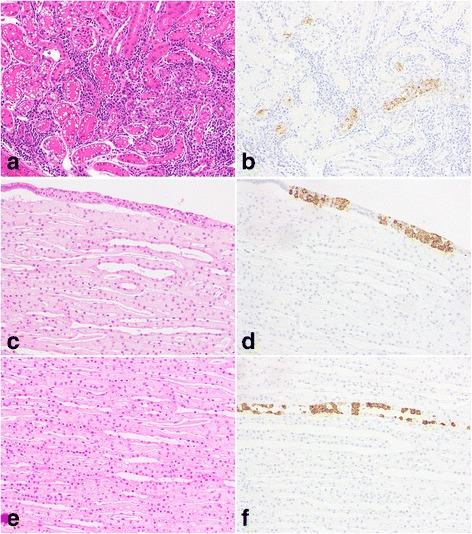
IHC examination of FmoPV-infected cases. **a** FmoPV RNA+ and Ab + positive case (ID of cat: N101). Severe infiltration of inflammatory cells into the interstitial tissue or renal tubules is observed (HE staining). **b** FmoPV RNA+ and Ab + positive case (ID of cat: N110). FmoPV antigens were demonstrated in the cytoplasm of renal tubular cells (IHC). **c** FmoPV RNA+ and Ab- case (ID of cat: N055). **d** FmoPV RNA+ and Ab- case (ID of cat: N055). Transitional cells in the renal pelvis are positive (IHC). **e** FmoPV RNA+ and Ab- case (ID of cat: N076). There are no pathologic lesions in this case (data not shown, Table 2). **f** FmoPV RNA+ and Ab- case (ID of cat: N076). Renal tubular cells are positive (IHC)

Histopathological examination was performed to detect FmoPV in cat renal tissues and to confirm the relationship between virus infection and tubulointerstitial nephritis by hematoxylin and eosin (HE) staining and immunohistochemistry (IHC) (Fig. 4). Among 29 cases that showed FmoPV RNA+ and/or Ab+, moderate to severe chronic interstitial nephritis was observed in 16 cases (Table 2 and Fig. 4). Mild infiltration of inflammatory cells into the renal tissues was observed in 10 cases. Three cases had no lesions. Other lesions including interstitial fibrosis, glomerulosclerosis, tubular microcystic change, proteinaceous casts and calcification were observed in some cases (data not shown). Among 71 FmoPV non-infected cats, moderate to severe chronic interstitial nephritis was observed in 29 cases. No lesions or mild infiltration of inflammatory cells into the renal tissues were observed in 25 and 15 cases, respectively. No statistically significant relationship could be confirmed between tubulointerstitial nephritis and the infection of FmoPV (Table 3, *P* > 0.05). However, a significant relationship was found between the presence of inflammatory lesions and the infection of FmoPV (Table 4, *P* < 0.01).

**Table 3:** The relationship between the grade of lesions and the infection of FmoPV

Infection of FmoPV	Grade of lesions				Total
	1^a^ (*n*)	2^b^	3^c^	4^d^	
FmoPV-positive	3	10	16	0	29
FmoPV-negative	25	15	29	2	71

^a^ No lesions

^b^ Mild infiltration of inflammatory cells

^c^ Moderate to severe TIN

^d^ et al

**Table 4:** The relationship between the presence of inflammatory lesions and the infection of FmoPV

Infection of FmoPV	The presence of inflammatory lesions		Total
	Existed (*n*)	Not existed (*n*)	
FmoPV-positive	26	3	29
FmoPV-negative	44	27	71

**Fig. 5:**
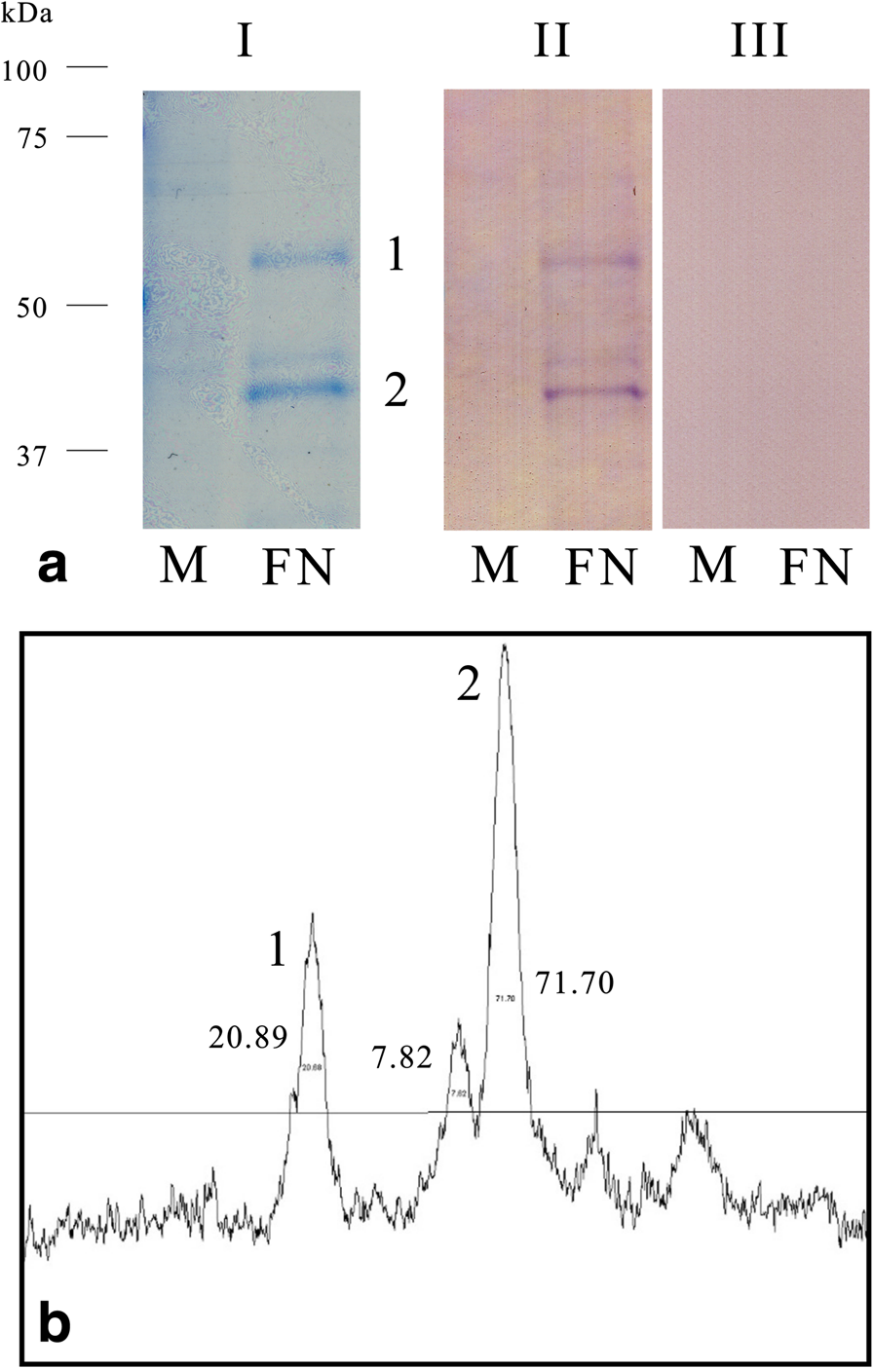
Analysis of recombinant FmoPV-N protein. **a** Coomassie Brilliant Blue (CBB) staining and Immunoblot. Two major bands were stained with CBB (M; mock, FN; FmoPV-N) (I). A large band corresponded to the entire FmoPV-N with a molecular weight of 59 kDa (1), while other smaller band may correspond to a truncated FmoPV-N with a molecular weight of 40 kDa (2). FmoPV antibody positive cat serum (N028) reacted to both two bands (II). FmoPV antibody negative cat serum (N004) did not react to mock and FmoPV-N protein (III). **b** Purity of the recombinant FmoPV-Ab. An image of the stained gel was analyzed using ImageJ 1.50i and it was shown that amount of the entire FmoPV-N and the truncated protein were approximately 21 and 72 %, respectively. The purity was estimated to be 93 %

To prepare rabbit antibody against FmoPV-N protein for the detection of FmoPV antigens, a recombinant FmoPV-N protein with his-tag at C terminal was expressed in Sf9 insect cells using a recombinant baculovirus. The recombinant FmoPV-N was purified by Ni-NTA agarose chromatography and was confirmed by SDS-PAGE (Fig. 5). Two major bands with a molecular weight of 59 and 40 kDa were observed by SDS-PAGE. A large band was consistent with the predicted size of the FmoPV-N, while a small one is thought to be N-terminal truncated FmoPV-N, since both of them were purified by Ni-NTA agarose chromatography. Since FmoPV-positive cat serum reacted to both two bands in immunoblot, N-terminal truncated FmoPV-N was considered to be immunogenic. The purified FmoPV-N protein was subcutaneously injected into three rabbits five times. When the serum antibody titers by IF test reached above 1:10,000, the rabbits were euthanized and the sera were collected. The rabbit antibodies reacted to the recombinant FmoPV-N protein in immunoblot (data not shown).

Immunohistochemistry (IHC) was performed using the rabbit FmoPV-N antibody to detect viral antigens in tissues. Among 29 FmoPV-infected cats, 19 cats showed FmoPV-N proteins in renal tissues by IHC (Table 2). However, only four cases out of the 19 cats were positive for FmoPV-N proteins in,the tubular cells of the renal cortex and the renal medulla (Fig. 4). Extensive inflammatory cells were infiltrated around the FmoPV-N positive renal tubular cells, indicating the cats were suffered from chronic tubulointerstitial nephritis (Fig. 4a and b). Although other cases also showed mild to severe infiltration of mononuclear cells in the interstitial tissues, the distribution of FmoPV-N proteins was limited in renal tubular cells or transitional cells of the renal medulla or pelvis, but not in the inflammatory lesions (Fig. 4e and f). In all of the cases, cytoplasms of renal tubular cells were positive for FmoPV-N proteins and there were no specific intracytoplasmic or intranuclear inclusion bodies (Fig. 4). Overall, it seemed likely that FmoPV infection might be associated with feline urinary tract diseases including CKD or LUTD, although it remained inconclusive whether FmoPV causes these diseases per se or acts as a helper or bystander.

## Discussion

In the present study, 22 out of 100 cats in Japan were shown to be FmoPV RNA positive in urine and/or kidney. Phylogenetic analysis of the FmoPV detected in Japan showed FmoPV was clustered in three groups. However, a phylogenetic relationship with geographic distribution of FmoPV in Japan and Hong Kong was not observed. Further, a significant relationship between FmoPV cluster and renal disease in the cat was not observed (data not shown). It is, however, necessary to compare the FmoPV sequences in cats from other regions in future to confirm this hypothesis. Recently, several partial sequences of FmoPV detected in cats in Germany were reported [5], however, we could not compare our data with them because the region of the sequences were not overlapped.

RNAs and proteins of FmoPV were detected in cat urine, sera and/or renal tissues. Anti-FmoPV Abs were also demonstrated in cat sera. FmoPV-infected cats in the present study were divided into three groups: RNA+/Ab+, RNA+/Ab- and RNA-/Ab+. Cats in the RNA+/Ab- group were considered to be in an acute phase of FmoPV infection. Cats in the RNA+/Ab + group were considered to be in either a subacute or a chronic phase of the infection. Since it is not confirmed that the nested R-PCR used in the present study detected all the FmoPV strains because of the genetic diversity of the viruses, thus it is possible that there are some false negative results in the RT-PCR. Since humoral immune response in addition to cellular immune response to the virus are thought to eliminate the virus from animals, cats in the RNA-/Ab + group were considered to be in a convalescent phase. Consequently, it appears that FmoPV could establish as an acute, subacute or chronic infection, and could be eliminated. Additionally, there were cases in which RNA of FmoPV was negative in urine but positive in renal tissues. Since approximately half of the infected cats (14 out of the 29 infected cats) were positive for viral RNA and Ab, it seems likely that cats are easily chronically or persistently infected with the virus. However, further long-term follow-up studies in FmoPV-infected cats are necessary to confirm this hypothesis.

**Fig. 6:**
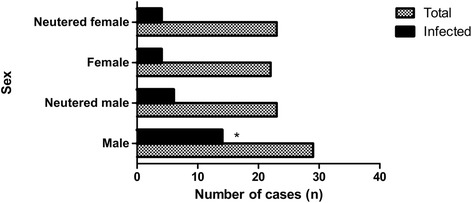
FmoPV-infected cats according to sex. The infection rate of FmoPV was significantly high in unneutered male cats (*P* < 0.005)

Cat samples were collected randomly from healthy cats and sick or wounded cats, including stray cats in Tokyo. No relationships among the clinical data, except age and sex, with the infection by FmoPV could be found. The infection rate of FmoPV was significantly high in unneutered male cats (*P* < 0.005) in this study (Fig. 6). These tendencies could be due to differences of behavior patterns, such as more aggressive or active behavior in unneutered male cats than neutered male cats and female cats, and such behavior might result in an increase in opportunity of infection with FmoPV from other infected cats. The transmission of FmoPV between cats could occur in veterinary hospitals, boarding kennels, breeding facilities, outdoors or at home. Given the high positive rate, it seemed that FmoPV might be maintained between their transmissible cats for a long time.

In a pathological examination, 25 out of 29 infected cats had renal lesions, from mild mononuclear cell infiltration to chronic tubulointerstitial nephritis. Half of them were mediate to severe cases. As a result of IHC, 19 out of 29 cats showed FmoPV-N protein in their kidney tissues. Four of the 19 cats showed severe lesions with FmoPV-N protein in the inflammatory lesions. The FmoPV-N protein-positive cells were identified to be renal tubular epithelial cells in the renal cortex, medulla and pelvis. FmoPV-N protein-positive mononuclear cells could not be found in this study, different from a report in Hong Kong [1]. These cases had RNA of FmoPV both in urine and renal tissues and relatively high IgG titer of 1: 640 to 20,480, except one case, representing the chronic phase. However, the IHC-positive sites were focally located, not distributed widely in the lesions.

In the other 15 cases, normal renal tubular cells or transitional cells of the renal medulla or pelvis were IHC-positive. No statistically significant correlations were found between the grades of lesions and the infection of FmoPV. However, a statistically significant relationship with the presence of inflammatory lesions and the infection of FmoPV was found, regardless of TIN. Consequently, no meaningful direct relationship between infection by FmoPV and chronic TIN was confirmed in this study. It might be plausible that FmoPV antigens in the lesions were already eliminated by host immune responses in the case of severe chronic TIN. A similar phenomenon was reported in other virus infectious diseases such as hemorrhagic fever with renal syndrome (HFRS) caused by hantavirus and severe acute respiratory syndrome (SARS) caused by SARS coronavirus (SARS-CoV) [19, 20]. In these cases, uncontrolled responses in cytokines and chemokines are considered to be responsible for the pathogenesis. In this regard, it would be necessary to analyze innate immune responses in cats with FmoPV.

It is well known that many viruses such as feline immunodeficiency virus, feline foamy virus, feline infectious peritonitis virus and feline leukemia virus can affect the kidneys of domestic cats, resulting in severe CKD including glomerulosclerosis, TIN, amyloidosis and pyrogranulomatous nephritis [21–27]. FmoPV is a recently identified virus, but it could affect the kidneys in infected cats. Thus, the existence or pathogenicity of FmoPV could have been underestimated.

In addition, a correlation between lower urinary tract disease (LUTD) and infection of FmoPV has been reported recently [5]. LUTD including urethral obstruction (UO) and interstitial cystitic (IC) is also an important and common disease in domestic cats [10, 11, 15–17, 28–30]. Feline calicivirus, feline herpesvirus type II and feline foamy virus have been detected in some cats with LUTD [21, 28–32]. In this study, lower urinary tract tissues or related clinical symptoms were not examined. However, it could not be excluded that FmoPV may also be related to LUTD, for two reasons: 1) only four cases had FmoPV antigens in inflammatory lesions of the renal cortex, even focal, 2) normal tubular cells of the renal medulla and transitional cells of the renal pelvis in proximity to the lower urinary tract were FmoPV antigen-positive.

Genetic diversity of FmoPV has been described extensively [3–5]. In Hong Kong, FmoPV seemed to be related to chronic TIN [1]. In Germany, it was detected in a LUTD case [5]. In addition, they detected FmoPV in a LUTD case showing 86 ~ 99 % homology compared to reported FmoPV [5]. At the moment, we could not detect FmoPV belonging to genetically diverged group found in Germany [5] among cats in Japan. We can not rule out the possibility that our RT-PCR and IHC could not detected these FmoPV RNA and N protein and their antibodies could not be detected using N proteins of FmoPV belonging to cluster C. However, it is unlikely since N proteins of paramyxoviruses in the same species are highly conserved and cross-reactive.

In our study, no clear relationship between FmoPV and chronic TIN or LUTD could be clarified although many types of FmoPV-infected cases are described.

It was suggested that FmoPV infection has a significant relationship with inflammatory reactions in the feline kidney and may be associated with urinary tract diseases. This was the first epidemiological report including seroprevalence and pathological examination of FmoPV among domestic cats in Japan. Considering the high infection rate and the genetic diversity of FmoPV, it seemed likely that FmoPV has evolved in Japan over a long period. Further retrospective and larger scale studies, including pathological studies, in other organs of cats are needed to elucidate the pathogenicity of FmoPV in cats.

## Conclusions

In this study, a high prevalence of FmoPV infection in domestic cats in Japan was revealed, and a significant relationship between FmoPV infection and inflammatory lesions in the kidneys was found. Further studies are necessary to elucidate the etiology of FmoPV in domestic cats. The serological and pathological examination established in this study might be useful for further surveillance.

## Methods

### Cat samples

Post-mortem urine (*n* = 100), sera (*n* = 100) and renal tissue samples (*n* = 100) were obtained from cats housed in the Tokyo Metropolitan Animal Care and Consultation Center. Cats in the center were carried by owners or captured in outdoors in Tokyo. Physical state and clinical symptoms were examined and recorded at the center. Cats randomly selected in the present study were healthy, sick or wounded, varying in age from new-born to 20 years old. The sex ratio of cats used in this study was similar.

### Detection of FmoPV

Total RNAs were extracted from cat urine (100 μL) and renal tissue samples (randomly selected three sites) using ISOGEN (Wako, Osaka, Japan) and a precipitation carrier (Ethachinmate, Wako), according to the manufacturer’s instructions. In the reverse transcription reaction, cDNA was synthesized by random primers (SuperScript R III First-strand synthesis supermix, Invitrogen, CA, USA) from the extracted RNA. Then, a part of L-gene sequence of FmoPV with 401 bp was amplified by nested polymerase chain reaction (PCR, 94 °C 5 min/94 °C 15 s, 55 °C 30 s, 68 °C 1 min/68 °C 5 min, first PCR : 40 cycles, second PCR : 20 cycles) using puRe Taq ready-to-go PCR beads (GE Healthcare, Buckinghamshire, UK) according to the previous reports [2, 4]. The primer sets used in the nested RT-PCR were previously reported [2]. A PCR product amplified from a FmoPV positive cat was purified and cloned into the pCR™ 4-TOPO® vector (Invitrogen) and used as a positive control of the nested RT-PCR. The sensitivity of the nested RT-PCR was approximately 10 copies /reaction (data not shown).

### Phylogenetic analysis

RT-PCR amplicons were separated by agarose gel electrophoresis and purified from the agarose gel using illustra™ GFX™ PCR DNA and Gel Band Purification Kit (GE Healthcare), according to the manufacturer’s instruction. The purified RT-PCR amplicons were used as templates for sequencing on an ABI 3130 Genetic Analyzer (Applied Biosystems) using a BigDye Terminator v3.1 cycle sequencing kit (Applied Biosystems, CA, USA) with the first and second PCR primers. Phylogenetic analysis was performed according to previous reports [4, 33, 34]. Briefly, phylograms were constructed using with the maximum likelihood method with the Kimura two-parameter model included in the MEGA6 package. The robustness of the resulting branching patterns was tested using the bootstrap method with 1000 replicates. In the analysis, the sequences of FmoPV (Hong Kong : M252A, 761U and 776U, Japan : SS1, OtJP001, MiJP003 and ChJP073, outgroups : CDV-Acc.No. AB476401.1 and Nipah virus-Acc.No. JN808863.1) deposited in the GenBank were included in the analysis. To support the result of phylogenetic tree, pairwise distances were analyzed by MEGA6 package and a distribution of pairwise distances was represented by histogram [18]. Statistically significant differences were calculated by Welch’s *t* test.

### FmoPV-N protein-expressing HeLa cells

Since the nucleocapsid (N) protein of morbilliviruses is highly conserved among isolates and has immunogenic epitopes [35–37], a full-length N protein of FmoPV (761U strain, JQ411014) was expressed in HeLa cells. A plasmid pMK-RQ containing FmoPV-N cDNA was chemically synthesized (Life Technologies, Tokyo, Japan). FmoPV-N cDNA with a *Bam*HI restriction site sequence at each extremity was amplified by polymerase chain reaction (PCR) from pMK-RQ containing FmoPV-N cDNA using an N-BamHI-F primer 5’-AGG ATC CAC AAT GTC TAG TCT ATT GAG G-3’ and N-BamHI-R 5’-TGG ATC CAG TTA TTT TAG AAG GTC AGT-3’ and cloned into a BamHI site of pKS336 expression vector [38]. The resultant vector, pKS336-FmoPV/N, was transfected into HeLa cells, and selected by DMEM medium containing 5 % fetal calf serum (FCS) and Blasticidin S Hydrochloride (2 μg/ml, Kaken Pharmaceutical. Co, Tokyo, Japan). The generated FmoPV-N-expressing HeLa cells were mixed with mock HeLa cells at 1:1, spotted on 14-well HT-coated slide glasses (AR Brown, Tokyo, Japan), air dried and fixed with acetone at room temperature for 5 min and stored at -80 °C until use. The antigen slides were used for the immunofluorescence test described below.

### Preparation of rabbit antibody against FmoPV-N protein

Recombinant FmoPV-N protein with a histidine (His)-tag at C-terminal was prepared by a baculovirus expression vector system to obtain rabbit antibody against FmoPV-N protein. First, to construct the expression plasmid, cDNA of FmoPV-N protein (761U strain, Accession No. JQ411014) was amplified by PCR using N-BamHI-F and N-BamHI-R primers and cloned into a transfer vector, pAcYM1-His-C. To construct the recombinant baculovirus containing FmoPV-N cDNA, the resulting transfer vector, pAcYM1-His-C-FmoPV/N, was co-transfected with a linearized baculovirus DNA, BaculoGold™ DNA (BD Biosciences, San Jose, CA) using X-treme 9 GENE Transfection solution (Roche, Mannheim, Germany) into Sf9 insect cells. The cells were cultured in Sf-900 TM II SFM Complete × 1 (Gibco, NY, USA) with 10 % FCS and kanamycin for 7 days at 27 °C. The medium and cells were harvested and centrifuged at 3000 rpm for 10 min at room temperature. The supernatants containing recombinant baculovirus-FmoPV/N were collected and saved at 4 °C. The cell pellets were used in SDS-PAGE to confirm the expression of his-tagged FmoPV-N protein. Baculovirus-FmoPV/N was infected to Tn5 cells to express his-tagged FmoPV-N protein. Infected Tn5 cells were harvested at 3 days post-infection and his-tagged FmoPV-N protein was purified by Ni-NTA agarose (QIAGEN, Hilden, Germany) and His-Bind® buffer kit (Novagen, San Diego, CA, USA). Purity of the purified FmoPV-N protein was analyzed by SDS-polyacrylamide gel electrophoresis (SDS-PAGE) followed by Coomassie Brilliant Blue staining (Bio-Safe TM Coomassie G-250 stain, Bio-Rad, CA, USA). Stained bands were quantified using ImageJ 1.50i [39, 40]. The purified FmoPV-N protein was subcutaneously injected into three rabbits five times. When the serum antibody titer checked by IF reached above 1:10,000, the rabbits were euthanized and sera were collected.

They reacted with FmoPV-positive renal tissues by IF, IHC and immunoblot, but did not react with FmoPV-negative renal tissues.

### Immunoblot

The purified recombinant FmoPV-N protein with His-tag was separated by SDS-PAGE and electrophoretically transferred to Immu-Blot PVDF membranes (Bio Rad, CA, USA). The membranes were blocked with Blocking One (Nacalai tesque, Inc. Kyoto, Japan) at 4 °C overnight and were incubated with cat sera (FmoPV-N Ab-positive; N028, FmoPV-N Ab-negative; N004) at a dilution of 1:500 at room temperature for 2 h. Then, the membranes were incubated with horse radish peroxidase-conjugated recombinant protein A/G (Purified Recomb ® Protein A/G, peroxidase-conjugated, Thermo Scientific, MA, USA) at a dilution of 1:1000 at room temperature for 1 h. The reaction of the antibodies were visualized with EzWestBlue (ATTO Corporation, Tokyo, Japan).

### Immunofluorescence (IF) test

IF was performed to detect antibodies (Ab) to FmoPV-N protein in cat sera. Cat sera were serially diluted in phosphate-buffered saline (PBS (-)) at a dilution of 1;40 to 1;20,480. They were applied to the wells of the antigen slides described above, and reacted for 1 h at 37 °C in a humidified chamber. After washing in PBS(-), goat anti-cat-IgG (H + L) conjugated with fluorescein (FITC) (1:1000, ROCKLAND, Gilbertsville, PA) was applied and incubated for 40 min at 37 °C. IF test was performed three times for each cat serum. Rabbit antibodies against FmoPV-N protein and goat anti-rabbit-IgG (H + L) conjugated with FITC were used as the positive control. Pre-sera from the same rabbits were used as negative controls. After washing in PBS(-), the slides were examined for staining patterns under a fluorescent microscope (Olympus BX51 and Olympus DP Controller 1. 1. 1.65 (Olympus, Tokyo, Japan). The Ab titers of tested cat sera were recorded as the reciprocals of the highest dilutions producing positive staining.

### Histopathological examination

Three sites including the renal cortex, medulla and pelvis were collected randomly and fixed in 10 % neutral formalin solution. Fixed tissues were embedded in paraffin, sectioned, and stained with hematoxylin and eosin (HE). For immunohistochemistry (IHC), deparaffinized sections were autoclaved at 121 °C for 10 min for antigen retrieval. Endogenous peroxidase activity was blocked by incubating sections with 3 % hydrogen peroxide in methanol at room temperature for 5 min. For the tissue blocking, sections were treated with 10 % skimmed milk in PBS(-) at 37 °C for 40 min. Then, rabbit antibodies against FmoPV-N protein (1:500 to 1:1000) were applied as primary antibodies at 4 °C overnight. Thereafter, sections were incubated with Envision System HRP-labeled polymer anti-rabbit (DAKO Japan, Kyoto, Japan) at room temperature for 40 min. Finally, the sections were visualized with 3,3’-diaminobenzidine tetrahydrochloride (Wako, Japan), and counterstained with Mayer’s hematoxylin solution (Wako). Degree of lesions was scored by severity of the lesions based on degree and extensiveness of tubulointerstitial nephritis or infiltration of inflammatory cells and other associated findings including interstitial fibrosis, necrotic changes, glomerulosclerosis and calcification. Scoring criterion + was defined as mild lesion with focal infiltration of small inflammatory cells. Scoring criterion ++ was defined as moderate lesion with infiltration of moderate inflammatory cells. Scoring criterion +++ was defined as severe lesions with severe infiltration of inflammatory cells. Distribution of FmoPV-N antigens in the renal tissues by IHC were also scored. Scoring criterion * was defined as FmoPV-N antigen positive cells focally in normal renal tissues, and criterion ** was defined as FmoPV antigens in several sites, while criterion *** was defined as extensive FmoPV antigen positive cells around lesions.

### Statistical analysis

Results are expressed as the number of cases. Statistical analyses were performed using StatFlex software (ver. 6, Artech Co., Ltd., Osaka, Japan). Significant differences between groups were determined by the Mann-Whitney *U*-test and ϰ^2^ test.
